# Pre-B acute lymphoblastic leukemia with t(1;19) in an adult initially presenting as hematuria and bilateral renal enlargement: a case report and literature review 

**DOI:** 10.5414/CNCS109113

**Published:** 2017-09-15

**Authors:** Jian Wu, Xiao-Ling Pi, Zhi-bin Ye

**Affiliations:** 1 Department of Pathology,; 2 Department of Nephrology, Gongli Hospital affiliated to Second Military, Medical University, and; 3 Department of Nephrology, Huadong Hospital affiliated to Fudan University, Shanghai, China

**Keywords:** ALL, acute renal failure, initial presentation, t(1;19)

## Abstract

Although pre-B acute lymphoblastic leukemia (ALL) is the most common type of renal leukemic infiltration; the renal infiltration with leukemia cells as the initial manifestation of leukemia is very rare. Translocation (1;19)(q23;p13) is one of the most common chromosomal abnormalities in patients with ALL and is observed in 5 – 6% of children with pre-B ALL. However, the incidence of t(1;19) in adults is lower, not exceeding 3%, and the prognosis of adult patients is usually poor. Herein, we report a 52-year-old female patient with pre-B ALL who initially presented as bilateral renal enlargement. The cytogenetic analysis revealed chromosomal abnormalities including t(1;19). The patient underwent three consecutive courses of chemotherapy with VDLP (vincristine, daunorubicin, L-asp, and prednisolone) and gained a short complete remission. Her kidneys recovered to normal size, and renal function returned to normal level. However, after complete remission for only 3 months, the patient exhibited resistance to consolidation chemotherapy and indicated evidence of marrow relapse. Although we increased the drug dosage and attempted to use a different protocol, she died of severe anemia and hemorrhage almost 10 months after she was first admitted. In conclusion, pre-B cell ALL is the most common type of leukemia to present with renal infiltration as the presenting sign. Because of the poor outcome of ALL, some new therapeutic approaches may improve the patients’ conditions.

## Introduction 

Symptoms of acute lymphoblast leukemia (ALL) usually present as fatigue, lack of energy, dyspnea, dizziness, bleeding, easy bruising, and infections arising from the expansion of leukemic cells in the bone marrow, peripheral blood, and extramedullary sites. Renal involvement is more common in children, especially during the late stage of the disease and in relapse cases [1]. However, involvement of skin, testicles, kidneys, joints, and bones is uncommon in adults [2]. The most common type of leukemia initially manifested as renal involvement is pre-B ALL. There are four reports in the English literature describing five cases of adult pre-B ALL presenting initially with renal involvement [3, 4, 5, 6]. There are some other types of leukemia which can also cause renal injury and acute renal failure [7, 8]. 

The t(1;19)(q23;p13) translocation is one of the most common chromosomal abnormalities in patients with ALL. In children with pre-B ALL, t(1;19) is observed in 5 – 6% of patients overall, whereas the incidence of t(1;19) in adults is lower, not exceeding 3% [2]. Herein, we describe for the first time an unusual case of pre-B ALL in an adult initially presenting as acute renal failure and bilateral renal enlargement that was associated with chromosomal abnormalities of t(1;19). 

## Case report 

**Figure 1.**
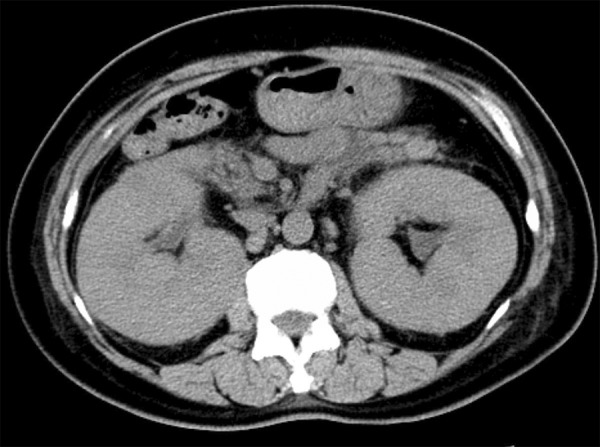
Abdominal computed tomography scan showing the bilateral enlarged kidney as well as retroperitoneal lymphadenopathy.

A 52-year-old woman was admitted with a 2-month history of lumbago and a 2-week history of gross hematuria. Recently, she complained that her hypertension was difficult to control in spite of antihypertensive drug treatment (Norvasc). Blood testing revealed the following: blood urea nitrogen (BUN) 34 mg/dL, serum creatinine (Scr) 2.72 mg/dL, and lactate dehydrogenase (LDH) 523 U/L. The complete blood count showed the following: hemoglobin (Hb) 85 g/L, white blood cell count (WBC) 15.1 × 10^9^ cells/L, and lymphocyte count 4.6 × 10^9^ cells/L. Urine testing revealed as follows: red blood cell count 291 cells/HPF, WBC 4 cells**/**HPF, protein ±, and occult blood +++. Abdominal computed tomography (CT) showed homogeneously enhanced soft tissues occupying the bilateral enlarged kidney as well as retroperitoneal lymphadenopathy (Figure 1). The patient also underwent a CT scan of her chest, but no valuable information was obtained. 

**Figure 2.**
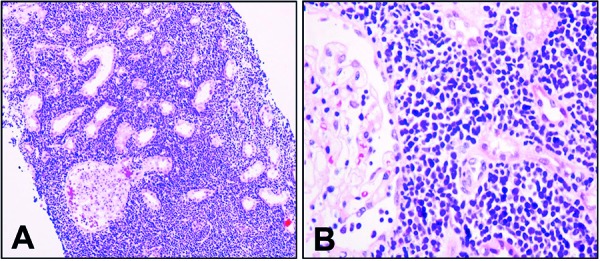
A: The kidney was diffusely involved with atypical small to medium blasts (H & E, ×200). B: The blasts exhibited minimal cytoplasm, a high nuclear: cytoplasmic ratio, convoluted hyperchromatic nuclei, and inconspicuous nucleoli (H & E, ×400).

**Figure 3.**
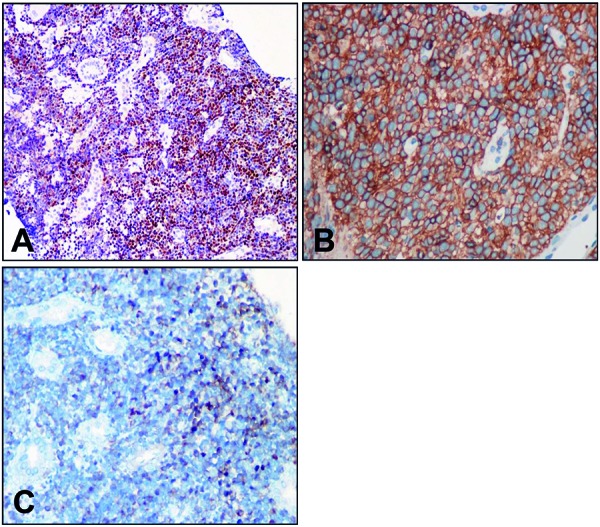
A: The blast cells were positive for TdT (×200). B: CD10 staining was positive in the blast cells (×400). C: CD79α staining was partly positive in the blast cells (×400).

A percutaneous renal biopsy indicated that the renal interstitium was diffusely infiltrated with atypical small to medium round cells (Figure 2A), which were characterized by minimal cytoplasm, a high ratio of nuclear to cytoplasm, convoluted hyperchromatic nuclei, and inconspicuous nucleoli (Figure 2B). Immunohistochemical staining revealed that the leukemic cells were positive for TdT (Figure 3A) and CD10 (Figure 3B), partly positive for CD79α (Figure 3C), and negative for vimentin, CD99, NSE, CD20, BCL-2, CD5, CD23, cyclin-D1, BCL-6, CD3, and MPO. A Ki-67 stain indicated a proliferation rate of 90%. The blasts were round or oval in shape and exhibited less cytoplasm. Intracytoplasmic vacuoles could be observed in some tumor cells. Other normal cells of the marrow, such as myeloid cells, erythroid cells, megakaryocytes, and platelets, were significantly reduced. The immunophenotypic surface markers of the leukemic cells were determined using immunofluorescence by flow cytometric analysis. The blasts, mature lymphocytes, granulocytes, and erythroblasts accounted for 72%, 8%, 14%, and 6% of nucleated marrow cells, respectively. The leukemic cells were positive for CD10, CD19, CD79α, HLA-DR, and TdT, and negative for CD20, CD33, and CD34. The features of leukemic immunophenotype supported the diagnosis of pre-B ALL with renal involvement. The patient’s cytogenetic analysis indicated that the chromosomal mutations involved chromosomes 1, 6, 9, 13, and 19, and the karyotypes were as follows: 45, der(19)t(1;19)(q23;p13.3), der(6;9)(p10;p10), I(9)(q10), der(13), (q12q14)del(13)(q22q34), 46. 

The patient started ALL induction chemotherapy (VDLP, vincristine, daunorubicin, L-asp, and prednisolone) for three consecutive cycles. During the first cycle of chemotherapy, the patient developed severe pancytopenia and tumor lysis syndrome. The patient also exhibited hyperkalemia, hyperphosphatemia, hypocalcemia, and hyperlithuria. Her blood pressure became hypotensive as a result of the metabolic acidosis and electrolyte disturbances. However, the patient’s condition was well controlled after the appropriate treatments, including supplying plasma and platelets, correcting electrolytes, stimulating marrow growth, and intravenous injection of anti-infection drugs. At the end of the first cycle of chemotherapy, her renal function returned to normal: BUN 11.4 mg/dL and Scr 0.87 mg/dL. Repeated marrow biopsies indicated an initial complete remission, and blasts accounted for only 0.2% of the total marrow cells. The patient’s kidneys returned to the normal size after the third cycle of chemotherapy. She was then treated with a consolidation chemotherapy protocol for another three cycles. However, the marrow biopsy after the sixth cycle of chemotherapy indicated evidence of marrow recurrence. Although we increased the drug dosage and attempted to use a different medical protocol, the patient exhibited resistance to chemotherapy and recurrence in the marrow. The last marrow biopsy after the seventh cycle of chemotherapy indicated that the tumor cells accounted for 80 – 90% of the total marrow cells. The latest laboratory test revealed the patient’s hemoglobin was 50 g/L, and the platelet count was 10×10^9^/L. She died with severe anemia and hemorrhage 10 months after she was first admitted to hospital. 

## Discussion 

Pre-B ALL is the most common type of leukemia initially presenting with renal involvement. The clinicopathological features of the five cases concerning adult pre-B ALLs with renal enlargement as the initial presentation and our case are listed in Table 1. The patients were middle aged and the age ranged from 22 to 53 years (median 43.3 years). Four were women, and 2 were men. The clinical presentations were not characteristic, and the patients complained of fever, body pain, and hypertension. Most patients showed abnormal renal function except for 1 patient (case 2), who had normal BUN and Scr value. Only half of patients (3 cases) showed anemia, and all patients had high WBC and LDH value. CT examination indicated that the renal tissues were involved by diffuse leukemic infiltration (4 cases) or focal infiltration (2 cases). Therefore, we suggest that WBC and high serum LDH value may provide important clues for the possible diagnosis of leukemia with renal involvement for patients manifesting with acute renal failure and renal enlargement. 

Pre-B ALL is morphologically indistinguishable from pre-B lymphoblastic lymphoma (pre-B LBL). The World Health Organization classification of tumors of hematopoietic and lymphoid tissues defines the two tumors as different clinical stages of the same biological entity. When manifesting as a tumor mass without any or minimal peripheral blood or marrow involvement, the tumor should be diagnosed as pre-B LBL. With extensive peripheral blood and marrow involvement, it is suitable to term the tumor as pre-B ALL. Therefore, marrow biopsy is substantial for the identification of them. 

TdT is restricted to lymphoid precursors, and CD19 and CD79a are highly specific markers for B lineage. In the present case, the blasts expressed TdT, CD19, and CD79a, which suggested that these cells were derived from the pre-B-cell lineage. 

The patient’s immunophenotype was consistent with the characteristic surface antigen profile of pre-B ALL with t(1;19), which includes CD19^+^, CD10^+^, CD22^+^, CD34^–^, and CD20^+/–^, whether the translocation is balanced or derivative [9]. Because other types of leukemia can also infiltrate renal tissue, it is important to differentiate pre-B ALL from T-cell ALL, minimally differentiated acute myeloid leukemia (AML), and the blastoid variant of mantle cell lymphoma. The positive expression of CD3 and CD5 is characteristic of T-cell ALL. The strongly positive myeloperoxidase (MPO) staining suggested the diagnosis of AML, and Cyclin-D1 is a useful marker for mantle cell lymphoma. 

Pre-B ALL also needs to be distinguished from other small round cell tumors occurring in the kidney. Ewing’s sarcoma/PNET usually occurs in the central nervous system or soft tissue which also can occur in the kidney in rare cases [10]. The positive expression of vimentin and CD99 in Ewing’s sarcoma/PNET can help to distinguish it from pre-B ALL. Renal carcinoid tumor is a rare neoplasm, and is composed of monomorphic round or polygonal cells with granular amphophilic to eosinophilic cytoplasm. The tumor cells demonstrated trabecular, ribbon-like, and gyriform patterns, and the test result positive for immune markers of chromogranin A and synaptophysin can help its differentiation from other analogous renal tumors [11, 12]. Primary renal neuroblastoma is also rare. Although it has some identical morphological features with pre-B ALL, it usually occurs in infants [13]. 

One of the most common translocations found in patients with ALL is t(1;19)(q23;p13). This translocation can alter the E2A gene on chromosome 19p13 in childhood B-ALL, leading to the formation of a fusion gene (E2A-PBX1) that encodes a hybrid transcription factor with oncogenic potential. The E2A-PBX1 fusion protein functions as a potent transcriptional activator and transforms several cell types in vitro, including fibroblasts, myeloid progenitors, and lymphoblasts [2]. The translocation t(1;19) had a balanced form, t(1;19), and an unbalanced form der t(1;19), and the initial response to chemotherapy and short-term outcome showed no difference between the balanced and unbalanced forms of t(1;19). 

The chromosome abnormalities might have important indicative roles for the patients’ prognosis. In children with pre-B ALL, the presence of translocations is associated with a significantly increased incidence of traditional high-risk features, and t(1;19) is largely responsible for the poor prognosis. However, a more intensive chemotherapy regimen can offset the negative impact of children ALL with t(1;19) [14]. With contemporary treatment, patients with the t(1;19) and E2A/PBX1 fusion have a favorable overall outcome but an increased risk of CNS relapse [15]. In adults with t(1;19), the prognostic relevance is not as good as in children. Most adult patients with t(1;19) usually failed to therapy within 1 year and do not respond to intensive therapy [16]. With the development of leukemogenesis, tyrosine kinase inhibitors like dasatinib may provide one hopeful treatment for adult ALL with t(1;19) [17]. 

In conclusion, pre-B cell ALL is the most common type of leukemia to present with renal infiltration as the presenting sign. The translocation t(1:19) is one of many possible reasons for the poor result of patients. Therefore, we should offer some new therapeutic approaches to improve the patients’ conditions. 

Written informed consent was obtained from the parents of the patient for publication of this case report and accompanying images. 

## Funding 

This work is supported by Shanghai Health Bureau grant (20134464). 

## Conflict of interest 

The authors declare that they have no conflict of interests. 

**Table 1.** Clinicopathological features of adult ALL with renal involvement as initial presentation in the cases reported in the English literature and in the present case.

No.	Author/Year	Sex	Age	Clinical presentation	Lab examination					Type of renal lesion	Treatment	Clinical outcome
					WBC	Hb	BUN	Scr	LDH			
1	Au et al. [3] (2000)	F	47	Fever, weight loss	8.9×10^9^/L	11.7 g/dL	9.2 mmol/L	179 µmol/L	764 IU/L	Diffuse infiltration	Na	CR
2	Au et al. [3] (2000)	F	44	Fever, weight loss	8.6×10^9^/L	10.4 g/dL	4.0 mmol/L	70 µmol/L	1,750 IU/L	Multiple cortical lesson	Na	CR
3	Su et al. [4] (2007)	M	42	Headache, facial, paralysis	34.1×10^9^/L	14.1 g/dL	12..1 mmol/L	274 µmol/L	Na	Diffuse infiltration	VP	CR
4	Zhou et al. [5] (2010)	F	53	Nausea, vomiting, fever	7.9×10^9^/L	7.8 g/dL	Na	255 µmol/L	5,63 IU/L	Diffuse infiltration	VDLP	CR
5	Gupta et al. [6] (2010)	M	22	Abdominal pain, vomiting, vertigo	8.9×10^9^/L	12.6 g/dL	14.3 mmol/L	304 µmol/L	764 IU/L	Multiple cortical lesson	Na	CR
6	Present case (2012)	F	52	Lumbago, hematuria, hypertension	15.1×10^9^/L	85 g/dL	15.2 mmol/L	240 µmol/L	523 U/L	Diffuse infiltration	VDLP	Relapse

WBC = white blood count; Hb = hemoglobin; BUN = blood urea nitrogen, Scr = Creatinine, Na = Not available; CR = complete remission; VP = vincristine, prednisolone, VDLP = vincristine, daunorubicin, L-asp, prednisolone prednisolone.
